# Supplementary material for: Studying the decontamination process of an irradiated beryllium reflector in a chlorine environment
Source: PLoS One. 2025 May 16;20(5):e0322723. doi: 10.1371/journal.pone.0322723 (PMC12083820; doi:10.1371/journal.pone.0322723)
Supplement: Attachment — Submitted filename: Response to reviewers.docx [file pone.0322723.s001.docx]

**PONE-D-25-00607**

**Studying the decontamination process of an irradiated beryllium reflector in a chlorine environment**

**PLOS ONE**

**Dear Dr. Baklanova,**

Thank you for submitting your manuscript to PLOS ONE. After careful consideration, we feel that it has merit but does not fully meet PLOS ONE’s publication criteria as it currently stands. Therefore, we invite you to submit a revised version of the manuscript that addresses the points raised during the review process.

Please submit your revised manuscript by **Mar 14 2025 11:59PM**. If you will need more time than this to complete your revisions, please reply to this message or contact the journal office at plosone@plos.org. When you're ready to submit your revision, log on to https://www.editorialmanager.com/pone/ and select the 'Submissions Needing Revision' folder to locate your manuscript file.

Please include the following items when submitting your revised manuscript:

•	A rebuttal letter that responds to each point raised by the academic editor and reviewer(s). You should upload this letter as a separate file labeled **'Response to Reviewers'**.

•	A marked-up copy of your manuscript that highlights changes made to the original version. You should upload this as a separate file labeled '**Revised Manuscript with Track Changes'**.

•	An unmarked version of your revised paper without tracked changes. You should upload this as a separate file labeled **'Manuscript**'.

If you would like to make changes to your financial disclosure, please include your updated statement in your cover letter. Guidelines for resubmitting your figure files are available below the reviewer comments at the end of this letter.

When submitting your revision, we need you to address these additional requirements.

1. Please ensure that your manuscript meets PLOS ONE's style requirements, including those for file naming.

2. Please update your submission to use the PLOS LaTeX template.

3. Thank you for stating the following financial disclosure:

 [The work was financed by the Science Committee of the Ministry of Science and Higher Education of the Republic of Kazakhstan within the framework of IRN BR21882185 “Research to support the creation and safe operation of a nuclear power plant in the Republic of Kazakhstan”].

Please state what role the funders took in the study.  If the funders had no role, please state: ""The funders had no role in study design, data collection and analysis, decision to publish, or preparation of the manuscript.""

If this statement is not correct you must amend it as needed.

Please include this amended Role of Funder statement in your cover letter; we will change the online submission form on your behalf.

4. We note that your Data Availability Statement is currently as follows: [All relevant data are within the manuscript and its Supporting Information files.]

Please confirm at this time whether or not your submission contains all raw data required to replicate the results of your study. Authors must share the “minimal data set” for their submission. PLOS defines the minimal data set to consist of the data required to replicate all study findings reported in the article, as well as related metadata and methods (https://journals.plos.org/plosone/s/data-availability#loc-minimal-data-set-definition).

For example, authors should submit the following data:

- The values behind the means, standard deviations and other measures reported;

- The values used to build graphs;

- The points extracted from images for analysis.

Authors do not need to submit their entire data set if only a portion of the data was used in the reported study.

If your submission does not contain these data, please either upload them as Supporting Information files or deposit them to a stable, public repository and provide us with the relevant URLs, DOIs, or accession numbers. For a list of recommended repositories, please see https://journals.plos.org/plosone/s/recommended-repositories.

If there are ethical or legal restrictions on sharing a de-identified data set, please explain them in detail (e.g., data contain potentially sensitive information, data are owned by a third-party organization, etc.) and who has imposed them (e.g., an ethics committee). Please also provide contact information for a data access committee, ethics committee, or other institutional body to which data requests may be sent. If data are owned by a third party, please indicate how others may request data access.

5. Please remove your figures from within your manuscript file, leaving only the individual TIFF/EPS image files, uploaded separately. These will be automatically included in the reviewers’ PDF.

6. Please review your reference list to ensure that it is complete and correct. If you have cited papers that have been retracted, please include the rationale for doing so in the manuscript text, or remove these references and replace them with relevant current references. Any changes to the reference list should be mentioned in the rebuttal letter that accompanies your revised manuscript. If you need to cite a retracted article, indicate the article’s retracted status in the References list and also include a citation and full reference for the retraction notice.

We look forward to receiving your revised manuscript.

Kind regards,

Mohammad Alrwashdeh

Academic Editor

PLOS ONE

**Dear Mohammad Alrwashdeh,**

Thank you indeed for your e-mail regarding the reviewers’ comments on our manuscript titled “Studying the decontamination process of an irradiated beryllium reflector in a chlorine environment”, referenced as “PONE-D-25-00607”. I would like to thank the reviewers for their careful reading of the manuscript and thoughtful comments.

I have carefully addressed all the comments and have incorporated the necessary revisions in the manuscript. A detailed point-by-point response to the reviewers’ comments is provided in the letter. Additionally, I have prepared and included the following documents as per the journal’s requirements:

1. **Response to Reviewers** – A detailed response addressing each point raised by the academic editor and reviewers.
2. **Revised Manuscript with Track Changes** – Highlighting all modifications made in response to the feedback.
3. **Clean Revised Manuscript** – A final version without tracked changes for publication consideration.
4. **Updated Financial Disclosure Statement** – The funding statement has been amended as follows:

*This research has been funded by the Committee of Science of the Ministry of Science and Higher Education of the Republic of Kazakhstan (Grant No. BR21882185). The funders had no role in study design, data collection and analysis, decision to publish, or preparation of the manuscript.*

5. **Data Availability Statement** – I confirm that all relevant raw data required to replicate the results of the study, including values for means, standard deviations, and graphical data, are provided within the manuscript. All relevant data are within the manuscript.
6. **Figures and References** – Figures have been removed from the main manuscript file and uploaded separately as individual TIFF image files. I have also carefully reviewed the reference list to ensure its completeness and correctness.

I hope that these revisions meet the journal’s expectations, and we sincerely appreciate the time and effort of the reviewers and editorial board in evaluating our work. Please let us know if any further modifications are required.

Here in the following table, I have listed the Reviewers’ comments and my corresponding responses.

I have also modified/corrected the manuscript accordingly.

I hope the revised manuscript will fulfill the standard required for publication in *PLOS ONE*.

Thank you very much in advance for your considerations and look forward to hearing from you soon.

Sincerely yours,

**Yuliya Baklanova**

|  | **Reviewer Comments** | **Author Response** | **Place in the Revised Manuscript** |
| --- | --- | --- | --- |
| **Reviewer #1**  **General Comments** | I have reviewed the article titled "Studying the decontamination process of an irradiated beryllium reflector in a chlorine environment". The article present an interesting study to decontaminate the beryllium used in nuclear reactor. My comments are as follows. | I sincerely appreciate your thoughtful review and constructive feedback.  Overall, I am grateful for your insightful comments, which have allowed the quality of the manuscript to improve. I hope that the revisions satisfactorily address your concerns. Below, I address each of your points in detail |  |
| **Reviewer #1**  **Comment #1** | There are a few typos in the article. For example, in abstract it is written "H3 including tritium" when it should be "including H3 tritium".  For the gamma radiation spectrum graphs, the ordinate and abscissa titles are not in English.  Page 2, 118mAg is wrong.  Similarly page 4, 60CoCl2 is not written correctly in 3rd paragraph. | I am sorry for the inaccuracy. I have corrected all typos, including the phrase in the abstract, as well as thoroughly the text of the manuscript.  I am sorry for the inaccuracy. The axis titles of all gamma radiation spectrum graphs have been translated into English for consistency. All corrected figures can be found at the end of the table.  The isotope notation on page 2, as well thoroughly the text of the manuscript has been corrected to **Ag^108m^**.  The chemical formula for cobalt chloride on page 4 has been corrected to **^60^CoCl_2_**. | **Abstract**  Page 1  Please, see corrected Figures:  Fig.1.tif, Fig.4.tif,  Fig.5.tif, Fig.6.tif,  Pages 2-5.  Page 3. |
| **Reviewer #1**  **Comment #2** | In figure 1, 614 and 723 keV peaks are labelled as Ag-110 but in all the other figures the peaks are labelled as Ag-108. | As you right pointed out, the correct isotope should be Ag^108m^, and I have now made the necessary corrections in Fig. 1 to ensure consistency across all figures.  All corrected figures can be found at the end of the table. | **Materials and Methods**  Pages 2, 4, 5 |
| **Reviewer #1**  **Comment #3** | In results and discussion, it has been mentioned that tritium chloride capture efficiency is only 3%. additional studies are needed to verify the "assumption" that this efficiency can be increased with contact time of gaseous tritium chloride with water and using multi-stage tritium chloride traps | I appreciate the reviewer’s attention to this point. In the manuscript, I have stated that tritium chloride absorption efficiency does not exceed 3% and suggested that efficiency could be improved by increasing the contact time of gaseous tritium chloride with water, employing bubbling devices, or using multi-stage tritium chloride traps.  In References below, the relevant calculations have been provided:  *Yu. Baklanova, А.Vurim, V.Kotov, А. Sitnikov, L. Chernova. Features of chloride technology for processing irradiated beryllium. Polzunovsky bulletin No. 3 2019.* [*https://doi.org/10.25712/ASTU.2072-8921.2019.03.016*](https://doi.org/10.25712/ASTU.2072-8921.2019.03.016) *(In Russian)*  The current tritium capture system was primarily designed for proof-of-concept demonstration, and future research will focus on optimizing the system parameters to enhance tritium retention.  Additionally, my future research, titled "Study of Corrosion Behavior of Austenitic Steel 12X18N10T in a Chlorine Medium," which has been submitted to the *Journal of Corrosion and Materials Degradation*, further explores this topic.  I hope that the findings from this ongoing research will provide a more comprehensive understanding of the corrosion mechanisms involved. | **Discussion**  Page 5. |
| **Reviewer #1**  **Comment #4** | Page 5, iron chloride transportation in the system, which kind of candidate materials you have in mind to avoid this problem. | I appreciate the reviewer’s question regarding potential materials to mitigate iron chloride formation. As noted in the manuscript, the formation of iron chloride results from the interaction of chlorine with stainless steel components, which leads to corrosion.  To address this issue, quartz glasses, as well as borosilicate glasses, and nickel-based alloys are a promising candidate for improving resistance to chlorine-induced corrosion. However, I acknowledge that this “assumption” requires further experimental verification to determine the coating’s long-term stability under operational conditions. | **Discussion**  Page |
| **Reviewer #1**  **Comment #5** | First line of introduction, "the beryllium loses it unique properties as a neutron moderator and reflector". I think this needs to be modified or relevant reference needs to be provided which shows that there is decreases in beryllium's moderation and reflection capabilities after irradiation | I have revised the introduction to provide a more precise explanation of the degradation of beryllium's neutron-moderating and reflecting properties under prolonged irradiation.  Specifically, I have included references to studies that demonstrate how neutron-induced swelling, helium and tritium accumulation, and impurity activation affect beryllium’s structural and neutron transport properties, ultimately impacting its performance in reactors. | **Introduction**  Page 1  **References**  Pages 7-9 |

**Fig 1. Gamma radiation spectrum of the irradiated beryllium sample.**

**Fig 4. Gamma radiation spectrum of the sediment sample on the reaction chamber before the filter inlet.**

**Fig 5. Gamma radiation spectrum of the sample from the filter.**

**Fig 6. Gamma spectrum of the sample taken from beryllium chloride storage.**

|  | **Reviewer Comments** | **Author Response** | **Place in the Revised Manuscript** |
| --- | --- | --- | --- |
| **Reviewer #2**  **General Comment** | The manuscript focuses on an important topic in nuclear waste management - the decontamination of irradiated beryllium reflectors using chlorination processes. The underlying methodology is systematic and based on established chemical principles, validated through experimental measurements. The authors provide detailed documentation of the developed chlorination process and the results obtained from their pilot installation. The implementation of this decontamination approach addresses a significant challenge in nuclear waste management, particularly given the increasing global inventory of irradiated beryllium. Taking this into consideration, I recommend that the manuscript be considered for publication in PLOS ONE, subject to the following revisions: | Dear Reviewer,  I sincerely appreciate your careful review of the manuscript and your insightful comments.  I am grateful for these detailed recommendations, which have allowed me to improve the clarity and scientific depth of the work. I hope the revisions sufficiently address your concerns and appreciate your support in improving the manuscript  Below, I provide my responses to each of your points: | - |
| **Reviewer #2**  **Comment #1** | A major limitation of this study concerns the experimental validation which is based on only one type of sample from the JMTR reflector. It would be important to justify why this sample is considered representative and how the results could be extrapolated to other types of irradiated beryllium reflectors. A discussion of potential variations expected with different types of samples would significantly strengthen the scope of the study. | I acknowledge the Reviewer’s concern regarding the representativeness of the JMTR reflector sample used in this study. The choice of this sample was based on its well-documented, certified irradiation history, operational conditions, and composition, making it a suitable case study for evaluating the chlorination-based decontamination process, according to Ref. 18. |  |
| **Reviewer #2**  **Comment #2** | The chosen operational parameters, particularly the temperature of 730°C and the 30 chlorination cycles, require more thorough justification. The authors should explain how these parameters were optimized and present the relative efficiency of each cycle. This information is crucial for understanding the robustness of the process and its potential for optimization. | I appreciate this suggestion and have revised the manuscript to better explain the selection of 730°C as the reaction temperature and the 30-cycle process. These parameters were determined based on prior computational experimental data [please see Ref. 17 and 24] and thermodynamic analysis, ensuring efficient chlorination while preventing excessive volatilization losses.  The following sentences inserted into the text of the manuscript.  The installation's performance is calculated based on the fact that the operation of the proposed device consists of two phases: active and passive. After heating each unit of the installation to operating temperatures is supplied into the reaction chamber during the active phase of operation. The hot beryllium chloride flow reaches the filter, gradually increasing the temperature of its working elements. The active phase ends when the temperature of the filter's working elements reaches 700°C.  During the passive phase, chlorine supply is stopped, and the direct-flow system is cooled with argon,  The selection of operating temperatures is based on the phase transition temperatures of the obtained chlorides [22, 25]: the boiling point of beryllium chloride is 500°C, and the melting point of cobalt chloride is 724°C. The duration of the active phase was determined by the time required to heat nickel rods by 200°C in a flow of hot beryllium chloride, which was 6 minutes, while the duration of the passive phase is 5 minutes. |  |
| **Reviewer #2**  **Comment #3** | The formation of iron chloride due to corrosion represents a significant technical challenge that is not sufficiently addressed. The authors should discuss in more detail possible alternatives in terms of construction materials, evaluate the impact of this corrosion on the installation's lifespan, and propose solutions to minimize this problem, particularly in the perspective of larger-scale application. | The iron chloride formation due to corrosion poses a significant challenge in maintaining the integrity of the installation. In response to the reviewer’s request for more details:  •In terms of alternative materials, I assume that quartz glasses, as well as borosilicate glasses, and nickel-based alloys may offer improved corrosion resistance, though further experimental validation is required.  •For large-scale applications, design modifications such as improved gas flow control and protective coatings may be necessary to mitigate material degradation.  •These points have been inserted into the revised manuscript to provide a more comprehensive discussion of long-term system stability. | **Introduction**  Page 1,  Lines 30-33.  Page 2,  Lines 8-10  Lines 17-20. |
| **Reviewer #2**  **Comment #4** | Regarding formal aspects, the manuscript requires several improvements: sections should be clearly numbered, figures lack detailed captions and some are of insufficient quality, particularly the gamma spectra. Error bars should be added to all graphs and results tables. The bibliography should be enriched with more recent references on beryllium decontamination methods.  These clarifications and additions would make the manuscript more complete and relevant to the scientific and industrial community. | Sections have been numbered for better organization.  Figure captions have been expanded to provide clearer descriptions of the data presented.  Gamma spectra figures have been improved, and higher-quality versions have been included.  Error bars have been added to tables to better represent measurement uncertainties.  The References has been updated with additional recent references on beryllium decontamination methods to enhance the manuscript’s relevance. | Page 5,  Line 30  Page 6,  Line 5, 15 |
|  |  |  |  |
